# Pathway analysis of dilated cardiomyopathy using global proteomic profiling and enrichment maps

**DOI:** 10.1002/pmic.200900412

**Published:** 2010-03

**Authors:** Ruth Isserlin, Daniele Merico, Rasoul Alikhani-Koupaei, Anthony Gramolini, Gary D Bader, Andrew Emili

**Affiliations:** 1 Banting and Best Department of Medical Research, University of TorontoToronto, ON, Canada; 2 Donnelly Centre for Cellular and Biomolecular Research, University of TorontoToronto, ON, Canada; 3 Department of Physiology, University of TorontoToronto, ON, Canada

**Keywords:** Cardiomyopathy, Gene expression, MS, Pathway analysis, Quantitation, Systems biology

## Abstract

Global protein expression profiling can potentially uncover perturbations associated with common forms of heart disease. We have used shotgun MS/MS to monitor the state of biological systems in cardiac tissue correlating with disease onset, cardiac insufficiency and progression to heart failure in a time-course mouse model of dilated cardiomyopathy. However, interpreting the functional significance of the hundreds of differentially expressed proteins has been challenging. Here, we utilize improved enrichment statistical methods and an extensive collection of functionally related gene sets, gaining a more comprehensive understanding of the progressive alterations associated with functional decline in dilated cardiomyopathy. We visualize the enrichment results as an Enrichment Map, where significant gene sets are grouped based on annotation similarity. This approach vastly simplifies the interpretation of the large number of enriched gene sets found. For pathways of specific interest, such as Apoptosis and the MAPK (mitogen-activated protein kinase) cascade, we performed a more detailed analysis of the underlying signaling network, including experimental validation of expression patterns.

## 1 Introduction

Heart disease is a leading cause of death, accounting for >30% of all deaths in 2005 in the US alone 1. In particular, heart failure stemming from diverse etiologies, including hypertension, long-term consequences of myocardial infarction, viral infection and genetic disorders, is an emerging epidemic 2. Although treatable, heart failure is often referred to as a silent killer since patients are diagnosed at an end-stage when it is too late to reverse the pathology. Finding molecular signatures to detect heart failure at an early, treatable stage prior to clinical presentation is vital to improving long-term survival outcomes. However, uncovering the causative mechanisms and predictive biomarkers remains a daunting task 3,4 due to the complexity of disease development.

MS/MS-based proteomic profiling is a promising approach for characterizing protein perturbations associated with cardiac disease 5,6. In dilated cardiomyopathy (DCM), the ventricle walls stretch and thin out causing the heart to enlarge and fail. In a recent study of a mouse phospholamban transgenic mutant model (PLN-R9C) of DCM 7, we used exhaustive shotgun sequencing to examine quantitative changes in global protein expression patterns in cardiac ventricular tissue at distinct time points representing discernible clinical phenotypes (early, mid-, and end stages) along the trajectory to overt cardiac failure. A generalized linear model identified 593 proteins significantly differentially up- or down-regulated across three time points in PLN-R9C mice relative to normal control littermates. This list was reflective of a shift in energy metabolism, and activation of specific cellular stress response cascades that lead to apoptotic signaling 8–10. However, by focusing on the most differentially expressed proteins across all time points simultaneously, only a partial list of the strongest biological signals was detected. New analysis methods are required to study stage-specific differences in expression accompanying disease progression and weaker, but still important, signals.

Transcriptomic datasets are traditionally analyzed by scoring gene expression differentiality (*e.g*. between disease and healthy states) after normalization of the raw mRNA expression data using statistical methods that consider technical noise and biological variability 11. Candidate genes are then defined by setting a threshold on some measure of differential expression 12. Since biological responses tend to be functionally coherent, over-representation analysis (ORA) can be used to detect statistically significant differential expression of functionally related “gene sets” 12. A “gene set” is a collection of genes defined *a priori* that share some attribute or feature such as annotation to a common pathway (*e.g*. cell cycle or insulin signaling). The resulting list of affected gene sets is often biologically more intuitive than the larger lists of differential genes. Numerous software tools are available to perform ORA 12, including FunSpec 13, GoMiner 14, FatiGO 15, DAVID 16, BiNGO 17 and ErmineJ 18. Most ORA tools typically only use Gene Ontology (GO) annotation 19 as a convenient source of gene sets, though some are being adapted to exploit more detailed network-level information (*i.e*. gene–gene interactions) that is increasingly available 12,20.

While we used ORA previously to find alterations in stress responses and metabolism that may underlie tissue remodeling and fibrosis in our DCM model, we only considered the most differentially expressed gene products and hypothesize that we have overlooked a wealth of additional more subtle and stage-specific biologically interesting patterns. To address this, we used the Gene Set Enrichment Analysis (GSEA) method 21 to perform ORA on all of the available expression value changes. GSEA analyzes a ranking of gene products according to a differentiality statistic (*e.g*. ratio of expression in disease *versus* control). Gene sets are then tested to see if members lie more toward the top or bottom of the ranking than expected by chance alone (*i.e*. majority of members of a gene set are coordinately up- or down-regulated). Thus, we expect to uncover additional biological trends in our PLN-R9C data using this method because it considers all genes, not just the top most differential, and can find significant and coordinated expression patterns at the gene set level even if the expression of the genes within the set is weak.

To aid in the interpretation of our GSEA analysis, we developed a method, Enrichment Map, to intuitively visualize and compare the results across time points. In comparison with our initial published study 7, adoption of a more powerful enrichment test together with a simplified graphical organization of the results enabled the identification of additional biologically relevant perturbations associated with DCM. For pathways of specific interest we performed a more detailed analysis, explicitly considering the underlying signaling network and experimentally validating expression patterns.

## 2 Materials and methods

### 2.1 Protein samples and quantification

We used proteomics data from our previously published PLN-R9C DCM study 7. Briefly, this data was collected from cardiac protein extracts collected from pooled ventricle tissue obtained from two distinct strains of mice, one a transgenic model expressing a dominant Arginine to Cysteine point mutation at position 9 in the phospholamban protein, which results in the presentation of DCM phenotypically similar to the human condition 22, and the other healthy littermates (strain FVB/N) as a control. Three time points were profiled, representing early-stage (8 wk), mid-stage (16 wk) and end-stage (24 wk) disease 7. Six thousand one hundered and ninety high stringency proteins were identified by nanoelectrospray LC-MS/MS and quantified by spectral counting 23. The spectral count mapping to a particular protein was summed to generate a total count *per* protein *per* sample. We supplemented our original data with 1072 proteins that were detected by MS but previously discarded because they were detected with only a single unique high confidence peptide. This re-analysis was motivated by the discovery that most of these represent small, but biologically important proteins (*e.g*. brain natriuretic peptide, a 121 amino acid protein that is a validated biomarker of heart failure 24). This resulted in a list of 7262 proteins used for the current analysis. To correct for length bias, *i.e*. larger proteins produce more peptides and so tend toward higher counts, the counts were divided by the number of expected tryptic peptides in a similar fashion as described by Lu *et al.* 25. Protein counts were further normalized across all experiments using local polynomial regression fitting (Lowess) to adjust for residual differences 7. As we now account for protein length during normalization by dividing spectral counts by the expected number of observable peptides for each individual protein, the weight of proteins with a larger than expected number of observable peptides was relatively reduced and those from smaller proteins increased. This allowed us to apply more sensitive filters to low molecular weight proteins that are nevertheless important in signal transduction and other pathways that may be perturbed during the development of DCM.

### 2.2 GSEA analysis

We used GSEA 21 to compute gene set enrichment after ranking proteins by differential expression in disease *versus* control. Traditional transcriptomics analysis uses various statistical tests to compare the two phenotypic classes including “signal-to-noise,” “*t*-test” and “ratio of classes,” but these standard tests assume the data are normally distributed whereas our R9C proteomics data are not, due to the under sampling nature of MS/MS spectral counting 26. We also wanted to use a statistic that indicates directionality, *i.e*. whether the protein is up- or down-regulated. Thus, we used the non-parametric KS test to rank the proteins because it makes no assumptions as to the underlying data distribution and is signed. Using this statistic, 164 proteins were significantly (*p*-value<0.05) differentially expressed at the early stage (8 wk), of which 69 proteins were putatively up-regulated and 95 proteins were down-regulated, while 652 proteins were significantly affected at the mid-stage (16 wk), of which 495 proteins were putatively up-regulated and 157 proteins were down-regulated. However, all proteins are ranked and input into GSEA.

GSEA was run using gene sets from diverse public sources (described below). Small (<=15 genes) gene sets were removed because these are more likely to appear significant by chance alone. Large (>500 genes) gene sets were removed because they are typically too general to usefully interpret. Filtering has the added benefit of reducing the problem of false discovery by multiple testing. For each analysis, 1000 gene set permutations were used to compute a false-discovery rate.

### 2.3 Gene set collection

GO annotation was collected from the August 2008 download of the org.Mm.edGO2ALLEGS Bioconductor package. GO annotation was up-propagated so that all genes annotated to children terms were also assigned to the parent terms and genes were mapped to Entrez Gene identifiers. All available GO annotations were used to maximize gene coverage. To further improve gene coverage, we also collected all available BioPAX formatted pathways from Reactome 27, HumanCyc 28, National Cancer Institute Pathway Interaction Database 29, Integrating Network Objects with Hierarchies Pathway Database (www.inoh.org), Biocarta (www.biocarta.com), Cellmap (cancer.cellmap.org) and Netpath (www.netpath.org). BioPAX is a standard data exchange format for pathway information (www.biopax.org). Additional curated gene sets were collected from the Molecular Signatures Database 21, the comprehensive Resource of Mammalian protein complexes 30 and Disease Hub (http://zldev.ccbr.utoronto.ca/∼ddong/diseaseHub/). Since the pathway resources, except Reactome, provide human pathway information only, putative mouse homologs were cross-mapped based on orthology (ftp://ftp.informatics.jax.org/pub/reports/HMD_HGNC_Accession.rpt). Conversion tables for RefSeq and Uniprot to human Entrez Gene were downloaded from Biomart 31.

### 2.4 Enrichment Map analysis

To increase the power and coverage of our analysis, we collected gene sets from multiple independent sources (see above). Unfortunately, this also increases the number of redundant or similar gene sets, which complicates interpretation of results. To overcome this challenge, we developed a novel visualization approach, Enrichment Map, which organizes gene sets in a more intuitive way and which is implemented as a plugin for the Cytoscape network analysis environment 32. Enrichment Map places similar gene sets near each other, which results in a more concise global view of enriched biological functions (many gene sets related to the same function are grouped, which simplifies their display). This map is a network of gene sets in which the nodes (circles) represent statistically significant terms and the links (edges) the degree of gene set overlap (*i.e*. multiple gene sets containing the same genes). An automated layout algorithm is used to place connected (*i.e*. similar) gene sets close together as clusters of terms describing related pathways, cellular processes or functions. Gene sets are linked if their overlap coefficient is >0.5 (*i.e*. gene sets share 50% or more genes). Software to construct and browse Enrichment Map is freely available (http://www.baderlab.org/Software/EnrichmentMap) (Merico, D., Isserlin, R., Stueker, O., Emili, A., Bader, G. D., Enrichment Map: A network-based method for gene-set enrichment visualization and interpretaion. 2010, submitted).

## 3 Results

### 3.1 Differentially expressed gene sets in DCM preceding heart failure

**Figure 1:**
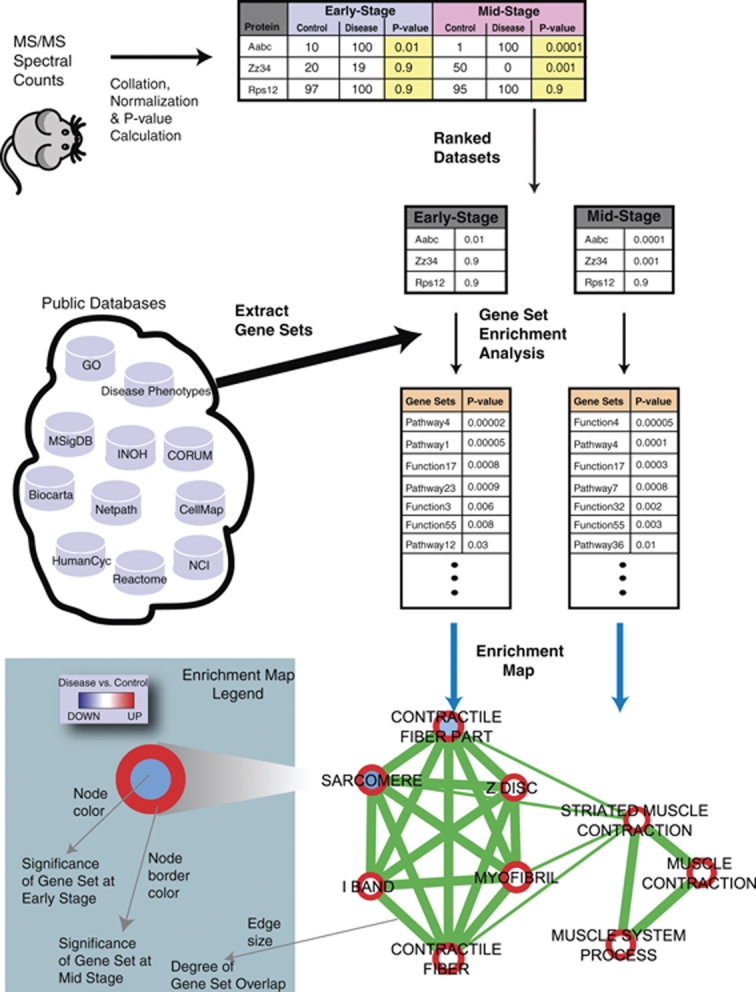
Enrichment analysis workflow. Outline of the processing of information from MS/MS data to Enrichment Map. First, spectral counts measured for each identified protein at two time points (early and mid-stage) in the PLN-R9C cardiovascular disease model and the healthy (wild-type) control 7 were normalized and ranked by *p*-value. The ranked protein list was then examined for significant over-representation of gene sets using the threshold-free technique of Gene Set Enrichment Analysis (GSEA). Gene sets were collected from a diverse set of public databases. Finally, the enrichment results were visualized to enable easy manual detection of global trends and hypothesis generation. A node in the Enrichment Map represents a gene set. Node color intensity represents the enrichment significance and the hue (blue/red) indicates whether a particular gene set is up- or down-regulated. Node size represents the gene set size and line thickness shows the degree of overlap (shared genes) between the two gene sets it connects. Two different enrichment experiments were simultaneously visualized to compare the enrichment results of the early- and mid-disease stages by mapping early-stage results to the node center (inner part) and mid-stage results to the node border (outer part).

The PLN-R9C mutant heart has a calcium flux imbalance due to the mutant (R9C) form of phospholamban which constitutively inhibits the SERCA ATPase responsible for calcium ion transport from the cytosol into the sarcoplasmic reticulum in muscle 22, which eventually leads to heart failure. In an effort to gain clinically useful insights into the causative basis of heart failure from the diverse proteomics patterns generated as part of our ongoing DCM profiling initiative 7,33–35, we developed a computational analysis workflow for interpreting global protein abundance data that combines a statistically principled gene set based enrichment analysis with an efficient graphical summary display for exploratory visualization (Fig. 1).

To generate ranked lists of differentially expressed proteins between the disease (*i.e*. PLN-R9C) and healthy (*i.e*. wild-type) hearts for analysis using our workflow, we normalized and scored our previously published mouse heart tissue protein abundance profiles, measured as spectral counts mapped with high confidence to cognate proteins by MS/MS (see Section 2). We then applied GSEA 21 to find gene sets that are enriched in differentially expressed proteins (see Section 2). To maximize protein coverage, we collected curated gene sets from 11 public repositories containing gene function annotations, pathways, protein complexes and disease signatures (Table 1). We focused our analysis on the patterns of differential protein expression at the two earliest time points available (8 and 16 wk) to uncover early- (*i.e*. pre-symptomatic) and mid-stage (*i.e*. clear evidence of cardiac functional defects but minimal morbidity) effects. This is in contrast to our original linear model analysis 7, which resulted a set of proteins differentially expressed across all three time points at once. Our re-analysis resulted in a marked improvement in both the variety and amount of significant gene sets that was returned compared to our original study 7. We found 266 enriched gene sets (*p*-value<0.01, false-discovery rate<0.1) for the early and mid-stages, compared to the 27 reported in the original paper (*p*-value<0.01) 7. This tenfold increase is presumably due to the increased number of gene sets used, the analysis of each time point separately (different enriched gene sets resulted at each time point) and the ability of GSEA to identify gene sets with weak, but coordinated, expression patterns 21. The original analysis identified an increase in cytoskeleton processes, muscle development, ER stress, protein degradation, unfolded protein response and apoptosis and a decrease in aerobic respiration and heart development, matching the DCM phenotype 7. Our analysis found all of these processes and many more, some of which are differentially perturbed at only one time point, including cell growth, immunity, translation, RNA processing, and more detailed views of metabolism and signaling.

**Table 1:** Publicly accessible curated gene set sources used in this study

Source	URL	Version
Reactome	http://www.reactome.org	Version 27 (December 2008)
Cancer Cell Map	http://cancer.cellmap.org/cellmap	May 22, 2006
Net Path	http://www.netpath.org	April 29, 2008
Integrating Network Objects with Hierarchies (INOH)	http://www.inoh.org	November 28, 2007
BioCyc	http://biocyc.org	March 9, 2009
NCI Pathway Interaction Database	http://pid.nci.nih.gov/PID/index.shtml	October 20, 2009
NCI Biocarta	http://pid.nci.nih.gov/PID/index.shtml	June 1, 2004
Molecular Signal Database (MSigDB) - c2 (pathways)	http://www.broad.mit.edu/gsea/msigdb	Version 2.5 (April 7, 2008)
Gene Ontology (GO)	http://www.bioconductor.org/packages/2.5/data/annotation/html/org.Mm.eg.db.html	August 2008
Disease Phenotypes	http://www.utoronto.ca/zhanglab/index.html	
Corum Mips Complexes	http://mips.gsf.de/genre/proj/corum	February 13, 2008

### 3.2 Enrichment Map visualization of global perturbations

**Figure 2:**
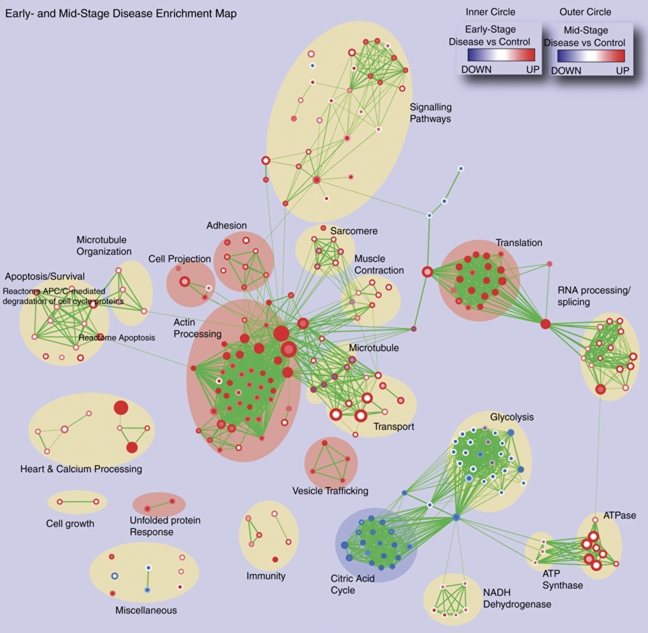
Processes perturbed in early- *versus* mid-stage DCM. Enrichment Map representation of the GSEA results obtained for the PLN-R9C transgenic mouse model of DCM *versus* wild type littermate controls at an early stage (8 wk, pre-symptomatic) and mid-stage (16 wk, reduced cardiac function but minimal morbidity) of heart disease. The inner circle is colored according to early stage onset, and the outer circle according to mid-stage disease. Node color and shading intensity represents the statistical significance of enrichment of a particular gene set.

To define a more concise picture of the pathways that are induced during the disease course, we visualized the early and mid-stage results as an Enrichment Map (see Section 2). A single integrated Enrichment Map was used to display the enrichment analysis results for both early- and mid-stage disease allowing direct comparison of the time points (Fig. 2) (Enrichment Map Cytoscape session available in Supporting Information at http://www.baderlab.org/Data/R9cEM). The node center (inner circle) color represents the enrichment obtained for the early time point, while the node border (outer circle) color reports the results for the mid-time point. From this diagram, it was easy to identify both similarities in the two time points, such as the uniform up-regulation of the actin remodeling machinery and protein translation (completely red circles) together with uniform down-regulation of the citric acid cycle (completely blue circles), and differences, such as the strong up-regulation of apoptosis, proteasome and RNA processing/splicing apparatus at the mid-stage (circles where one part is white and the other is colored). These differences likely represent a physiological response of the cardiomyocytes during the disease progression. For instance, the changes in energy metabolism (glycolysis, citric acid cycle and NADH dehydrogenase) shows evidence of a known shift in energy usage from more efficient aerobic respiration at early stage to less efficient anaerobic respiration at later stages, reminiscent of the Warburg effect seen in fast growing cancer cells 36. This also shows a limitation of our analysis, as energy metabolism is post-translationally regulated by many factors, including intracellular calcium, which is increased in PLN-R9C. Some of the changes (citric acid cycle) are expected, whereas others require more follow-up (initial glycolysis down-regulation, NADH and ATP synthase up-regulation). Up-regulation of many processes, including protein translation and RNA processing/splicing, are consistent with compensatory cardiomyocyte growth, associated with cardiac distension. By mid-stage, the effects of these stress responses appear to become detrimental, with the PLN-R9C mouse displaying extensive thinning of the ventricular wall, presumably due to an extensive loss of cardiomyocytes 7.

A novelty compared to our previous study is the difference in time-behavior of multiple processes, such as cytoskeleton control pathways. Changes in the sarcomere structure, the main contractile apparatus of the myocyte, are known to be preceded by actin remodeling 37. Actin-based cytoskeletal mobilization ultimately leads to cardiac remodeling 38, which is clinically evident as an enlarged heart size with a distended shape and contractile dysfunction (reduced fractional shortening and contraction force) 38. From the Enrichment Map, we see a consistent up-regulation of actin remodeling machinery at early- and mid-stage disease, whereas microtubule and sarcomeric up-regulation is only present at mid-stage disease. This highlights a potential difference in timing or coordination of these remodeling processes. Thus, the Enrichment Map significantly eases visual comparison of global trends in major cellular systems as a function of disease progression.

### 3.3 Exploring the apoptotic network

**Figure 3:**
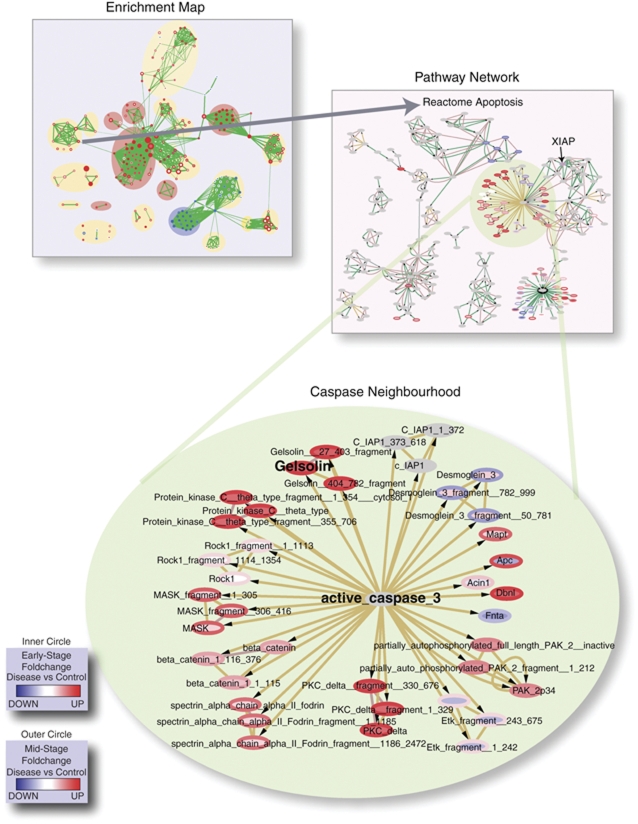
Activation of apoptotic signaling *via* caspase 3 and gelsolin. Consecutive zoom-ins of the Enrichment Map gene set cluster representing terms related to cellular apoptosis. Individual protein nodes represented in the pathway network are shown for the caspase neighborhood. Proteins are colored according to the expression ratio of condition *versus* control at the early (inner circle) and mid-stages (outer circle) of disease.

The enrichment map clearly showed apoptosis as a key element in the transition from an early-stage compensatory hypertrophy response (*i.e*. enhancing cardiac output) to mid-stage dilation, which precedes fibrosis and ultimately heart failure 8. Since many of the enriched gene sets originally came from pathway databases, which curate detailed protein interaction relationships, we were able to create a network view of the enriched apoptosis gene set from the Reactome pathway database, showing differential protein expression, using Cytoscape 32 (Fig. 3). Given that the transgenic PLN-R9C model has disrupted calcium flux within myocytes, an interesting active molecule in the pathway, gelsolin, stood out (Fig. 3). Gelsolin is a well-studied calcium regulated mediator of actin filament assembly and disassembly that was previously identified as a target of caspase-3-mediated apoptosis 39 and has previously been implicated in human DCM 40. Given that loss of gelsolin in a knockout mouse line 41 results in reduced apoptosis in response to myocardial infarction (artery ligation), which normally induces severe hypertrophy and dilation, the up-regulation of gelsolin (and other functionally related proteins) we detect suggests a causal connection to both the ventricular remodeling that precedes dilation (Fig. 2) and the increased apoptosis observed during disease progression 7. Gelsolin is one of many factors downstream of caspase-3 that are progressively up-regulated from early to mid-stage. Conversely, negative apoptosis related signaling factors appeared to be down-regulated. Most notable is an initial up-regulation at early stage, followed by down regulation at mid-stage, of the ubiquitin-protein ligase XIAP, a well-known inhibitor of apoptosis 42 (Fig. 3). Again, this is consistent with the overall gene set output showing a progressive increase in apoptosis during tissue remodeling and dilation.

Linked to apoptosis in the Enrichment Map is another large cluster representing up-regulation of the cell cycle at mid-stage disease. Although the gene set names are indicative of cell cycle events (*i.e*. Reactome_APC/C-Mediated degradation of cell cycle proteins), examination of the genes indicates this cluster is dominated by the proteasome complex, which is involved in multiple processes (including apoptosis and cell cycle). The ubiquitin-proteasome machinery is involved in the targeted cleavage and degradation of signaling proteins and has been linked to apoptotic cell death and the unfolded protein response previously seen in R9C 43. There are, however, conflicting reports as to whether the proteasome is up- or down-regulated in cardiac dysfunction 43. From our current analysis, we see a clear up-regulation of proteasome levels at mid-stage disease connecting to apoptosis in the Enrichment Map. The relationship between these two processes can be better gleaned from a more detailed mechanistic representation of the underlying gene sets.

### 3.4 Uncovering novel signaling pathways

**Figure 4:**
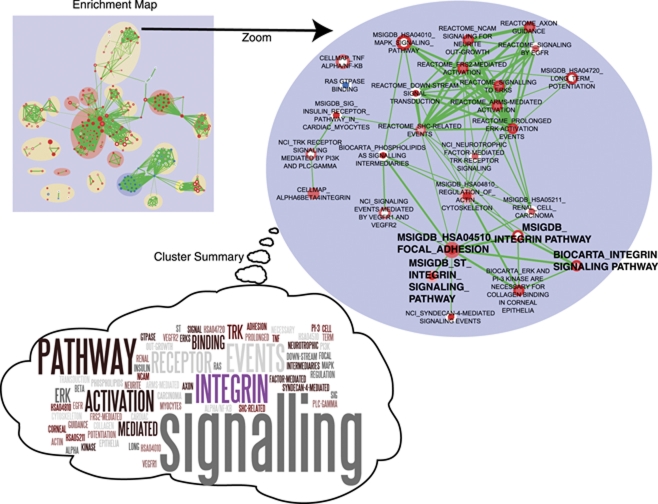
Signaling cluster and integrin signaling. Zoom-in of the Enrichment Map gene set cluster representing signaling pathways enriched at the early and mid-stages of heart failure. A summary description of the cluster was visualized as a “term cloud” using Wordle (http://www.wordle.net/) derived from the text descriptions of all gene sets. Term size indicates its frequency; thus, large terms best summarize the cluster (*i.e*. signaling pathways). Specific terms related to the integrin pathway are highlighted within this cluster and in the network.

A more sparsely connected cluster of multiple regulatory processes was found to be up-regulated to varying degrees at early- and/or mid-stage disease (Fig. 4). This grouping represents an assortment of interlinked pathways originating from different annotation databases. Integrin signaling is one of the more enriched pathways (highlighted in Fig. 4), with representations from three independent data sets linked *via* a focal adhesion term, which is consistent with the role of integrins as cell-adhesion receptors 44. Integrins also play a key role in sensing and transmitting mechanical load in cardiomyoctyes 45, connecting the extracellular matrix to intracellular signaling and the contractile apparatus (as can be seen by the connections between integrin signaling and actin cytoskeleton regulation in the enrichment map). In DCM, this process is involved in modifying the core contractile machinery to compensate for impaired calcium handling 45.

**Figure 5:**
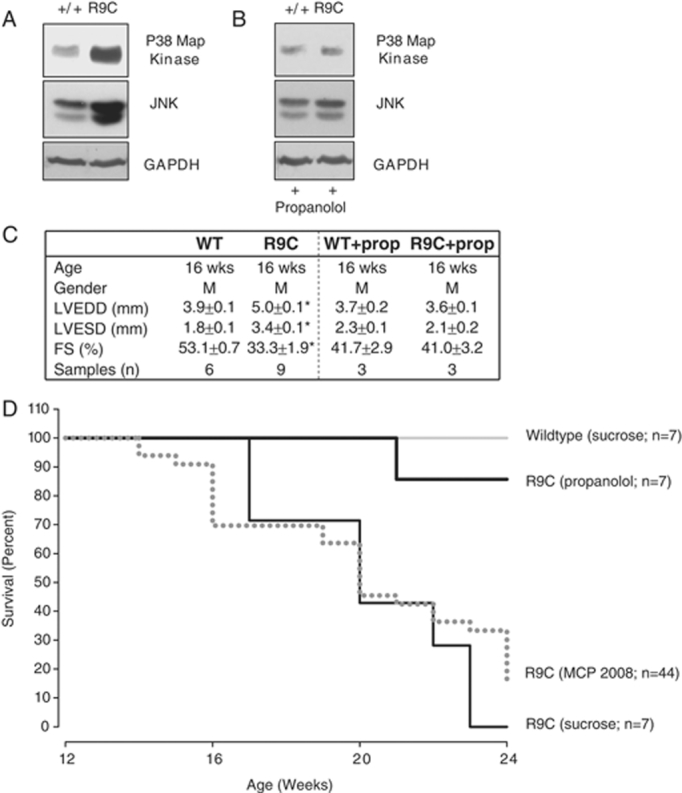
Reduced mortality and decreased MAPK activation with propanolol. (A) Cardiac cellular lysates from 16-wk-old mice were collected and analyzed for MAPK pathway activity (indicated by JNK expression and phosphorylation of p38 Map kinase), and *versus* a control (GAPDH). MAPK pathway is overactive in PLN-R9C mice. (B) Treating mice with propanolol reduces activity of MAPK pathway at 16 wk in PLN-R9C mice compared to wild type. (C) Sixteen-wk-old mice were subjected to M-mode echocardiography and left ventricular end diastolic dimension (LVEDD), left ventricular end systolic dimension (LVESD) and fractional shortening (FS) were assessed. Propanolol treatment reduces LVEDD, LVESD and fractional shortening to wild type levels. (D) WT and PLN-R9C mice were treated with/without propanolol (0.5 g/L in drinking water) starting at 8 wk of age. Mortality was monitored in all groups at 16 wk. Cardiac lysates and tissues were obtained and analyzed as previously described 7. Antibodies used: phospho-p38 – BD ♯612281 from BD bioscience and SAPK/JNK – mAb ♯9258 from Cell Signaling.

The MAP kinase signaling cascade is also prominently up-regulated at mid-stage and represented by multiple terms, including “Signaling to ERKS” (REACT_12058.1), “Prolonged ERK activation events” (REACT_12005.1) and “MAPK signaling” (KEGG:HSA04010) (Fig. 4). The MAPK (mitogen-activated protein kinase) signaling pathway, and more specifically up-regulation of p38 (MAPK14) 46 in rat myocytes, has been shown to induce heart dilation. Similarly, the down-regulation of the p38 47 or JNK (MAPK8) 48 catalytic subunits in transgenic mouse models followed by stress induced through aortic banding has been previously shown to induce cardiac hypertrophy leading to heart failure. In-depth analysis of the components in the MAPK signaling gene set from KEGG revealed that a handful of significantly up-regulated proteins and many additional weakly up-regulated factors were obtained by proteomic profiling. We therefore decided to focus on this group for targeted follow-up experiments since members of the pathway, such as p38 and JNK, have been previously linked to either hypertrophy or dilation depending on the direction of their differential expression. We examined the activation levels of the two key downstream effectors of the MAPK pathway, p38 and JNK, as indicators of pathway activity. As predicted from the GSEA results, both p38 and JNK show elevated activity in 16-wk-old PLN-R9C mutant mice as compared to wild-type controls (Fig. 5A), even though these proteins were not significantly up-regulated as measured by the KS statistic.

To further investigate the role of the MAPK pathway in mediating the progression to heart failure, we administered the beta blocker propanolol to PLN-R9C mice, which is commonly used clinically for treating heart failure 49 and can result in the reduction of MAPK-dependent pathway activation 50. Beta blockers function initially as negative ionotropic agents, decreasing the strength of muscle contraction thereby reducing energy requirements and wall stress 50. As demonstrated in Fig. 5B, both p38 and JNK returned to near wild-type levels at 16 wk after administration of propanolol starting at 8 wk. Further, phenotypic examinations (Fig. 5C) and the mice survival curves (Fig. 5D) also confirmed nearly complete rescue of PLN-R9C defect upon treatment with propanolol. These results imply causal participation of MAPK signaling, whose activation was missed in our initial proteomic assessment based on simple ORA analysis 7.

## 4 Concluding remarks

Like other groups, we have been investigating the causal basis for progressive DCM using an integrative profiling approach incorporating data from multiple relevant sources to generate a thorough, yet concise picture of the underlying functional disturbances over time. By applying GSEA to the early and mid-time points of DCM progression using a large and diverse set of pathways and functional annotations with an Enrichment Map display, we demonstrated how proteins ranked by relative expression in cardiac tissue in our PLN-R9C mouse model can be converted into a global view of processes changing over the course of heart disease progression, starting from pre-symptomatic pathology to DCM. These additional analyses have revealed novel functional connections, both between individual gene products and across biological pathways and broader systems, that were missed previously using simple ORA analysis 7. Our new method also more clearly shows processes affected in common, or uniquely, at the early- and mid-disease stages. These ranged from widespread effects on central metabolism and cytoskeletal remodeling to more specific perturbations in apoptotic, integrin and MAPK signaling.

While many of the gene sets, such as metabolism and actin remodeling, have been previously recognized in heart disease studies, there are still unanswered questions as to their mechanistic contributions to cardiac disease 51. The biological significance of the metabolism shift is suggested by recent publications indicating that it leads to a critical tipping point in the heart where energy reserves are not sufficient to maintain function which ultimately leads to failure 52. Our re-analysis highlights an early increase in energy demands by the heart manifested in the up-regulation of NADH Dehydrogenase and ATP synthase. This up-regulation is only detectable at an early stage when there is minimal phenotypic indication of any contraction defect, but disappears at mid-stage disease once the heart has already begun to fail, adding additional weight to the above tipping point interpretation.

The power of our method is that it can quickly identify general processes that are interesting and then enables a more detailed study, as can be seen from our analysis of apoptosis and MAPK signaling. This approach is flexible and can be applied to other datasets. As other high-throughput studies of DCM are conducted and gene set curation efforts continue, a more complete network will be generated, providing improved understanding of the underlying molecular basis for progressive heart failure in human patient populations. On-going efforts to improve the coverage of pathway data, encompassing transcriptional regulation by microRNAs and transcription factors 53–55, will likely provide the basis for more robust and informative computational analysis at the gene set and gene-interaction level. Increased protein coverage by MS and more sensitive methods will further expand the number of enriched gene sets, which may otherwise be missed due to too few differential proteins being present. The framework proposed here constitutes an ideal staging ground for more advanced computational tools supporting visualization, analysis and hypothesis generation for protein expression data. Although challenging, using pathway analysis to decipher the mechanism of a complex disease such as DCM facilitates the development of a more coherent molecular understanding of DCM etiologies and potentially other cardiovascular diseases that lead to heart failure.
